# Cultural adaptation process of six stigma assessment scales among Kannada speaking population in South India – CORRIGENDUM

**DOI:** 10.1017/gmh.2025.14

**Published:** 2025-02-18

**Authors:** Harshitha H Annajigowda, Gurucharan Bhaskar Mendon, Anish V. Cherian, Syed Shabab Wahid, Brandon A. Kohrt, Nicolas Rüsch, Sara Evans-Lacko, Elaine Brohan, Claire Henderson, Graham Thornicroft, Santosh Loganathan

**Keywords:** cross-cultural, adaptation, discrimination, assessment tools, mental illness

Upon publication of this article the author, Nicolas Rüsch, was misspelled as Nicolas Rüesch

The article has been updated to reflect this correctly.
