# Supplementary material for: Cultural adaptation process of six stigma assessment scales among Kannada speaking population in South India
Source: Glob Ment Health (Camb). 2025 Jan 14;12:e8. doi: 10.1017/gmh.2024.84 (PMC11810757; doi:10.1017/gmh.2024.84)
Supplement: Annajigowda et al. supplementary material [file S2054425124000840sup001.docx]

**Illustration of process of expert review and synthesis.**

| **Scale** | **Forward translated item** | **Concern raised by the experts** | **Alternatives suggested by experts** | **Finalized item** |
| --- | --- | --- | --- | --- |
| ASTA | “*I am inclined* to feel I fail to reduce stigma” | Discrepancy in the translation | It makes me feel…. (*bhavane moodisuthade*)  It feels like… (*anisuthade*)  I feel that... (*nanage anisuthe*) I think I have failed… (*naanu sothidene yenisuthade*) | *I feel that...* |
| SDS* | How willing would you be to have a person with mental illness as part of your microfinance, *community forest, or other community based organization?* | Inappropriate to the target community | …Garden and other community work  ….Community dependent work  ….Community organizations, *chit funds*, *gram panchayats,* women self help groups, and other community based organizations | ….Community organizations, *chit funds*, *gram panchayats,* women self-help groups, and other community based organizations |
| SSS* | *Prejudice* against people with mental illness will affect many areas of my life | The translated word for prejudice was flagged as complex to understand | Misconceptions  Notion  Wrong understanding | *Misconception* against people with mental illness will affect many areas of my life |
| DISCUS* | Have you been treated *unfairly*… | The translated word for ‘unfair’ was not colloquially used word. | Inappropriately  Mistreated  Unjustly | Have you been treated *unjustly…* |
| MAKS & RIBS* | *Mental health problems*… | The translated item was not colloquially used word | Mental illness  Mental disorders/diseases  Mental health problem | *Mental health problems* |

* the items were in target (Kannada) language. For ease of reader’s understanding, we have kept the English meaning. Although the English items may all seem the Kannada translations vary significantly.

**Cultural equivalence criteria:**

| **Criteria** | **Description** | **Examples** |
| --- | --- | --- |
| **Comprehensibility (Semantic equivalence)** | Is the item understandable in the language of the proposed respondents? Are local idioms used that the target groups will understand? | Selecting terminology for 'mental health' that is not technical and will be easily understood by lay respondents; selecting terminology for 'stigma' that will be easily understandable; consider adding examples when terms are difficult to understand. |
| **Acceptability (Semantic & content equivalence)** | Would participants using the tool be uncomfortable in any way using the tool? Are questions acceptable by local moral and religious standards? Would there be a strong social desirability bias to answer yes or no in response to this question. | Changing mental health terminology that may be especially negative, pejorative, or stigmatizing to more neutral terminology (e.g., changing 'crazy person' (*hucha*) to 'person living with a mental illness' (*manasika arogya samasye iruvavaru*)); adjusting for social desirability (e.g., 'would you be willing to marry a person with mental illness' to 'would you be willing to have a member of your family marry someone with a mental illness'. |
| **Relevance (Content equivalence)** | Is the description relevant in the local culture? Are the descriptions of stigma or mental illness relevant to the cultural group and the living circumstances? | Changing examples to fit context (e.g., adding/changing names of religious places & festivities of regional importance) Sharing a plate of food is normal (e.g., Ethiopia) vs. less common (e.g., India), then adjust wording to fit stigma accordingly (Ethiopia: 'would share a plate of food with someone with mental illness'. vs. India: 'would you be willing to eat a meal prepared by someone with mental illness'); |
| **Response Set (Technical equivalence)** | “What is the best method of assessment for this question?” Review the instructions, wording of the question, and the scoring, then discuss what might be a cultural source of difference in how the tool is used and scored. | Changing statements to questions and modifying response options, e.g., 'I am willing to buy food from someone with mental illness' with 'agree' / 'somewhat agree' / 'don't agree' to 'How willing are you to buy food from someone with mental illness' with 'fully willing' / 'somewhat willing' / 'not willing'. |
| **Completeness (Semantic, criterion, and conceptual equivalence)** | Does the culturally adapted version capture the same concepts as the original concept related to stigma and mental illness? For example, does the adapted version capture a process that is assumed to be stigmatizing and discriminating in that context? Is this related to beliefs and stereotypes of mental illness (e.g., mental illness is contagious, people with mental illness are violent, people with mental illness cannot be cured). | Adapt question so that they reflect local components of stigma related to ignorance, prejudice, and discrimination. |

**Illustration of process of cultural equivalence review.**

| **Scale** | **Forward translated Item** | **Concerned criteria** | **Finalized form** |
| --- | --- | --- | --- |
| **SSS** | I am able to stand the obstacles and meet the demands posed by misconceptions about people who have mental illness. | Completeness & comprehension | I am able to cope with the stigma and discrimination against people with mental illness |
| **SDS** | How much are you willing for someone you know to marry person who has a mental illness? | Relevance | How willing are you to have someone with mental illness marry someone related to you? |
| **SDS** | How willing are you to invite a person with mental illness to religious ceremonies or festivals held in a mosque / church or other place of worship? | Relevance | How willing are you to invite a person with mental illness to religious ceremonies or festivals held in a temple, mosque / church or other place of worship? |
| **DISCUS** | Have you been treated unfairly in dating or intimate relationships? | Acceptability & comprehension | Have you been treated unjustly in love or intimate relationships? |
| **MAKS** | Schizophrenia | Completeness & comprehension | Added transliterated word along with the target language word – *icchita vikalate (schizophrenia)* |
| **ASTA** | I think, when working with patients…. | Acceptability | I think when working with people with mental illness... |

**Example of adapted cognitive probe framework (Willis & Artino, 2013).**

| Type of cognitive probe | Example (Item - People with severe mental health problems can fully recover) |
| --- | --- |
| Comprehension/Interpretation | What does this question mean to you?  Based on this question, can any words be made simpler? |
| Paraphrasing | In your own words can you repeat the question you heard? |
| Confidence judgement | How sure are you about that answer? |
| Recall | What made you to answer in this way?  Do you know anyone with severe mental health problem fully recovered/not recovered? |
| Specific | Why do you say people with severe mental health problem do not recover? |
| General | Was that easy to answer?  You were using the term ‘mental illnesses.’ Is this term easier to understand than the term ‘mental health problems?’ |

.
